# Need for Swift Diagnosis of Primary Angiitis of Central Nervous System: A Case With Focal Motor Seizures of Hand Progressing to Aphasia

**DOI:** 10.7759/cureus.10803

**Published:** 2020-10-05

**Authors:** Saeed Arif, Shaheer Arif, Jahanzeb Liaqat, Wasim Wali Muhammad, Abdur Rahim Palwa

**Affiliations:** 1 Neurology, Pak-Emirates Military Hospital, Rawalpindi, PAK; 2 Neurology, Combined Military Hospital, Multan, PAK; 3 Radiology, Armed Forces Institute of Radiology and Imaging, Rawalpindi, PAK

**Keywords:** primary angiitis of the central nervous system, focal motor seizures, digital subtraction angiography, motor aphasia

## Abstract

Primary angiitis of the central nervous system (PACNS) is a rare disorder and difficult to diagnose. The time period from the presentation of the patient until a diagnosis can be protracted, which may result in further progression of the disease and poor patient outcomes. It has immensely varied symptomatology, further confounding swift diagnosis. We present a case of a 33-year-old male who had focal motor seizures with intact awareness of the right upper limb for three weeks prior to presenting to our hospital with acute right hemiparesis. One month into hospital admission, the patient developed complete motor aphasia while being investigated for the cause of multiple ischemic brain infarcts. The patient underwent immunosuppression along with plasmapheresis and pulse steroid therapy started prophylactically on suspicion of PACNS, which was subsequently confirmed by brain biopsy. Disease remission was achieved with rituximab.

## Introduction

Primary angiitis of the central nervous system (PACNS), although being a rare disease but can lead to high morbidity and mortality if not promptly managed. PACNS has a reported incidence of 2.4 per 1,000,000 person-years. There is a male predominance in reported cases. This disease can occur in almost any age group, with the median age of 47 years at the time of diagnosis [1].

PACNS has a kaleidoscopic clinical presentation with the most common presentation of patients exposed to headache with stroke, transient ischemic attack, cognitive impairment, and focal neurological deficits being [2]. Patients may present with an atypical clinical picture as well [3]. The histopathology of PACNS consists of Langerhans and foreign body giant cells, lymphocytic vasculitis, or necrotizing vasculitis. The classic picture presents granulomatous inflammation, which occurs in less than 50% of cases [4]. All these factors make the diagnosis of PACNS very difficult, particularly when a patient presents with unusual presentation and brain biopsy is not possible.

We demonstrate a patient with PACNS, who had focal onset motor seizures of the right hand as the initial manifestation of the disease, and presented to our hospital with acute right hemiparesis. Brain biopsy was only carried out after an unexpected second attack, which caused motor aphasia.

## Case presentation

A 33-year-old male patient without significant past medical history presented with acute right-sided hemiparesis involving the right arm and leg for eight hours. Initially, during this event patient felt tingling sensations in the right hand, which was followed by paresthesia in the unilateral right arm, followed by weakness in the same arm to the extent that patient was unable to raise his arm and even unable to hold any object in the right hand. Later, weakness involved the unilateral right lower limb, and the patient became unable to walk without support.

Before this, the patient had been experiencing focal onset motor seizures with intact awareness in the unilateral right upper limb, particularly the right hand, for the last three weeks. Seizures used to start with jerky movements of the right thumb and index finger and later spreading to the whole hand and wrist, with fast but small amplitude flexion and extension movements. These events used to occur for less than one minute before terminating without any intervention. He never had any kind of head trauma and never lost consciousness before or during these episodes.

The patient denied having any headache, visual symptoms, dysarthria, dysphagia, and palpitations during the current episode. He also denied having fever, weight loss, night sweats, or rash over the body, including the face. He was a non-smoker and never used any drugs for recreational purposes. Past medical history was non-revealing for hypertension, diabetes mellitus, cardiac pathologies, and chronic infections. To the best knowledge of the patient, family history for any kind of demyelinating neurological disorders and chronic rheumatologic or immunological disease was unremarkable.

On physical examination, the patient was conscious, oriented, and was comprehending all commands. Vital signs included heart rate 77 beats/minute, respiratory rate 21 breaths/minute, and blood pressure 130/85mmHg, without a postural drop on standing from a supine position. Neuropsychological assessment revealed normal higher cognitive functions. The neck was supple, and the kerning sign was unremarkable. All the cranial nerves were functioning normally. On the medical research council scale, power in the right upper limb was 2/5 while in the right lower limb was 3/5. Reflexes were slightly brisk on the right side. The planter response was extensor in the right foot. However, the left half of the body was normal. All sensory modalities were intact bilaterally except for slightly reduced fine touch sensation and two-point discrimination over the right arm. Examination of the chest, abdomen, and lungs was unremarkable. Moreover, the retina did not reveal any gross lesion on the slit-lamp examination.

**Figure 1:**
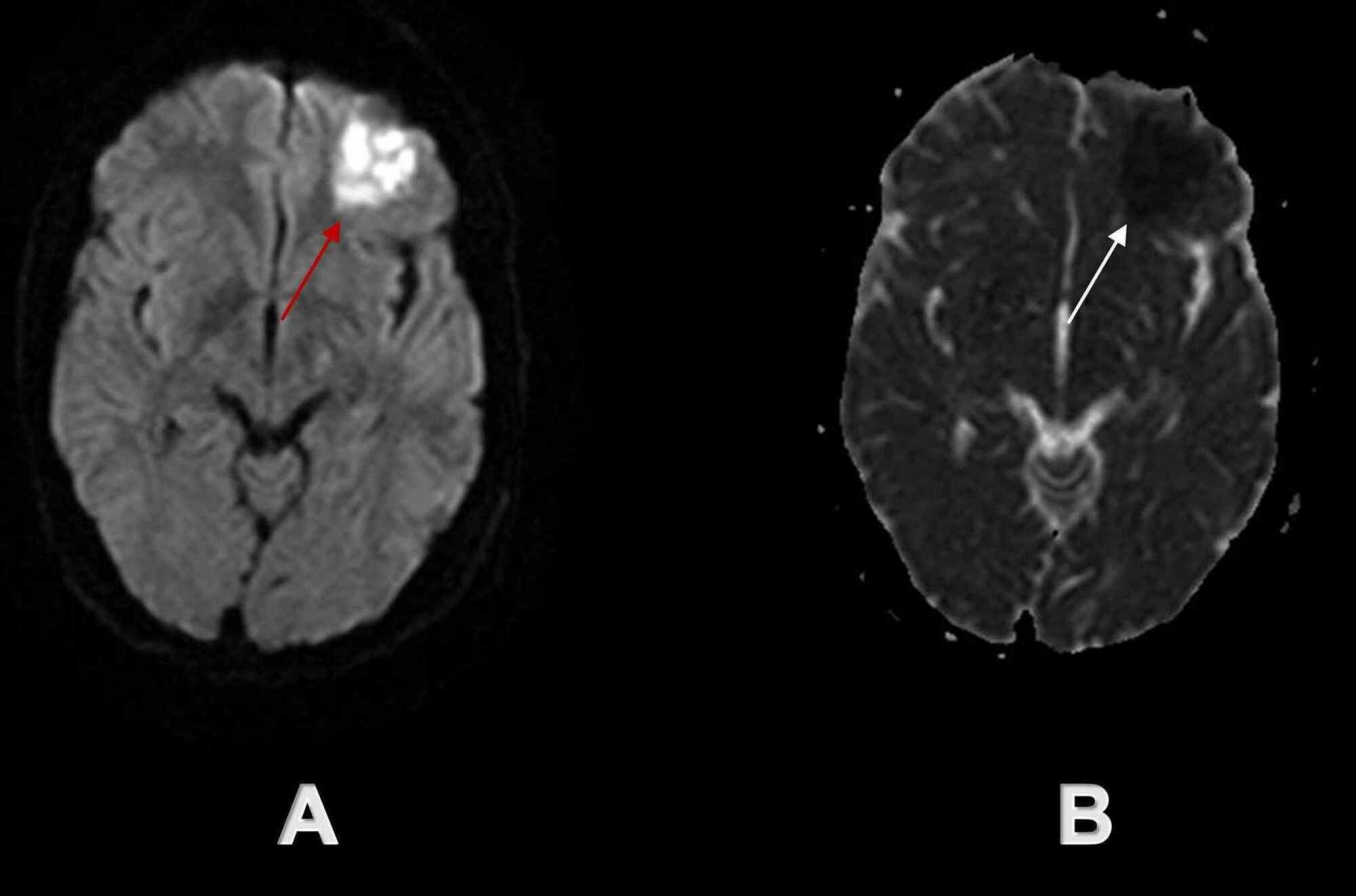
Initial brain MRI demonstrated acute ischemic infarct in the left frontal lobe The red arrow on axial DW sequence (A) and white arrow on axial ADC sequence (B) demonstrate acute ischemic infarct in the left frontal lobe. DW - diffusion-weighted; ADC - apparent-diffusion-coefficient

**Figure 2:**
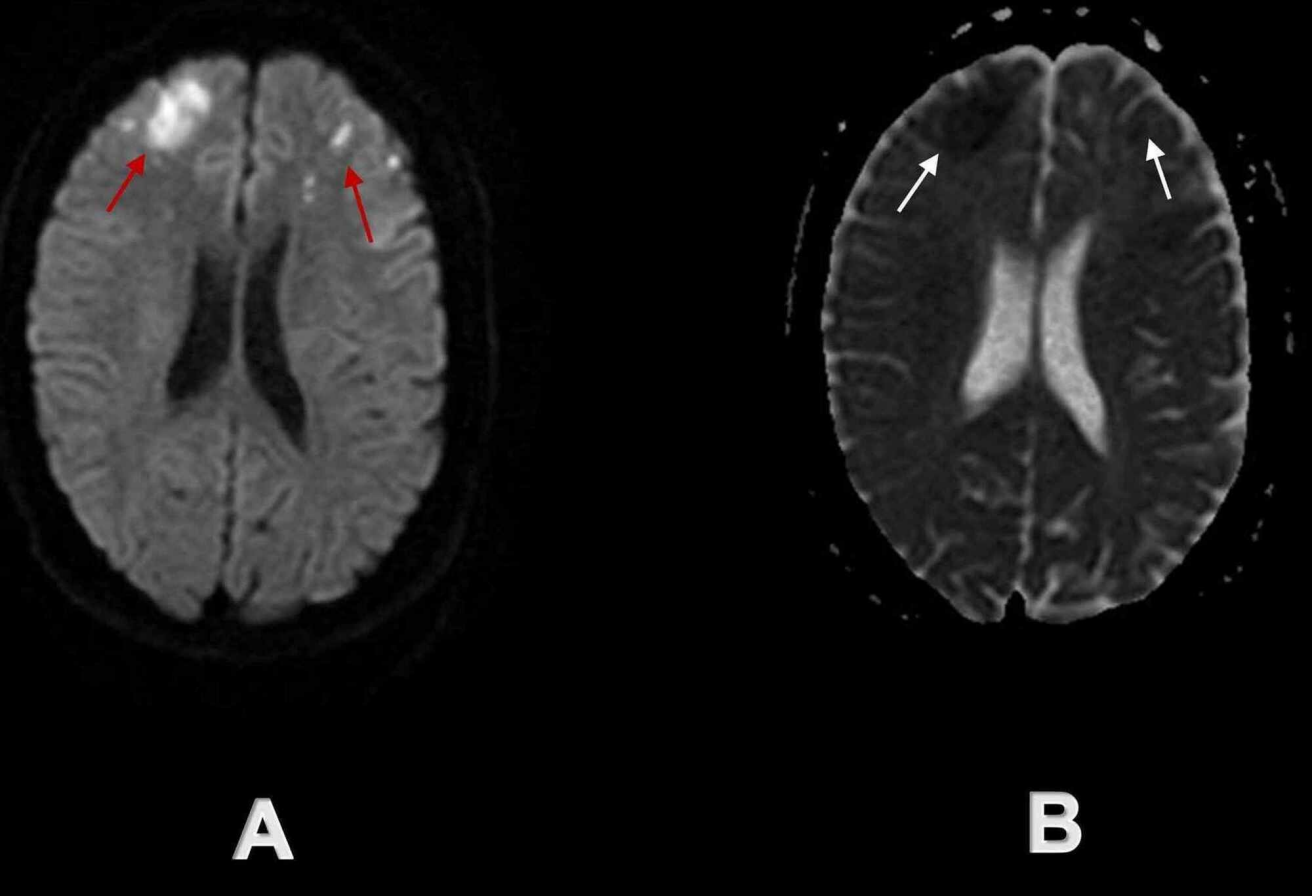
Initial brain MRI demonstrated multiple acute ischemic infarcts in bilateral frontal lobes Red arrows on axial DW sequence (A) and white arrows on axial ADC sequence (B) demonstrate multiple acute ischemic infarcts in bilateral frontal lobes. DW - diffusion-weighted; ADC - apparent-diffusion-coefficient

**Figure 3:**
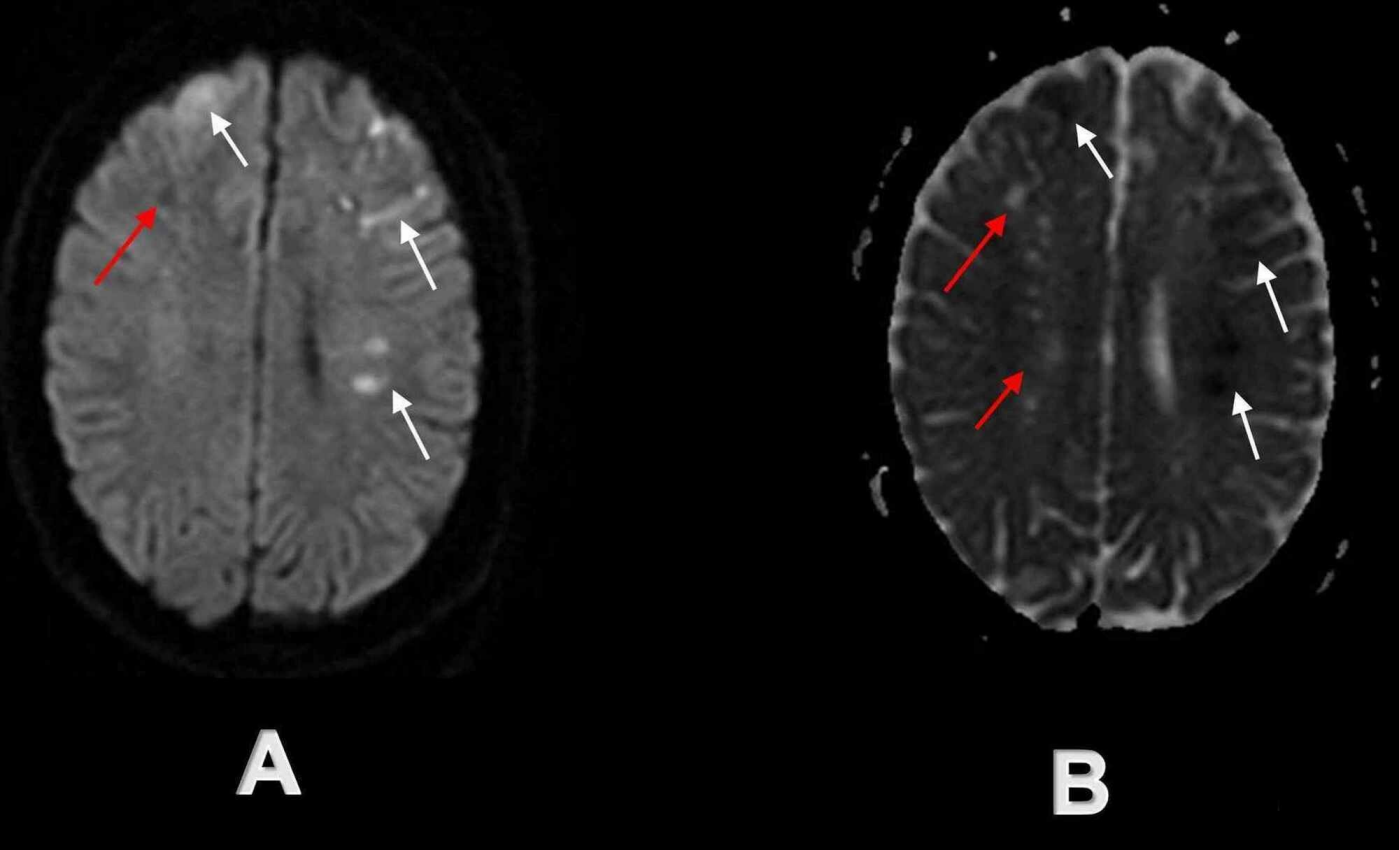
Initial brain MRI demonstrated acute and chronic ischemic infarcts in bilateral frontoparietal regions White arrows demonstrate multiple acute ischemic infarcts on axial DW sequence (A) and axial ADC sequence (B) in the bilateral frontal lobe and left parietal lobe, while red arrows demonstrate simultaneous chronic ischemic infarcts on axial DW sequence (A) and axial ADC sequence (B) in the right parietal lobe and bilateral centrum semi-ovale. DW - diffusion-weighted; ADC - apparent-diffusion-coefficient

**Figure 4:**
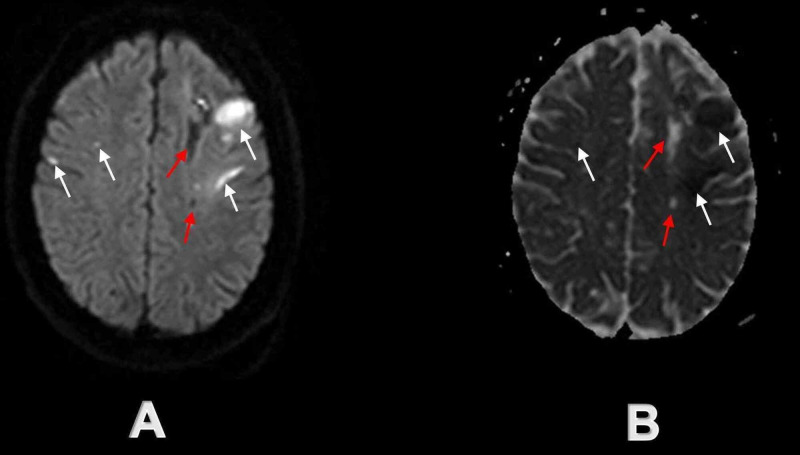
Initial brain MRI at the time of admission demonstrating acute and chronic infarcts in bilateral high frontoparietal lobes White arrows demonstrate multiple acute ischemic infarcts on axial DW sequence (A) and axial ADC sequence (B) in bilateral high frontoparietal lobes, while red arrows demonstrate simultaneous chronic ischemic infarcts on axial DW sequence (A) and axial ADC sequence (B) in the centrum semi-ovale mostly on the left side. DW - diffusion-weighted; ADC - apparent diffusion coefficient

**Figure 5:**
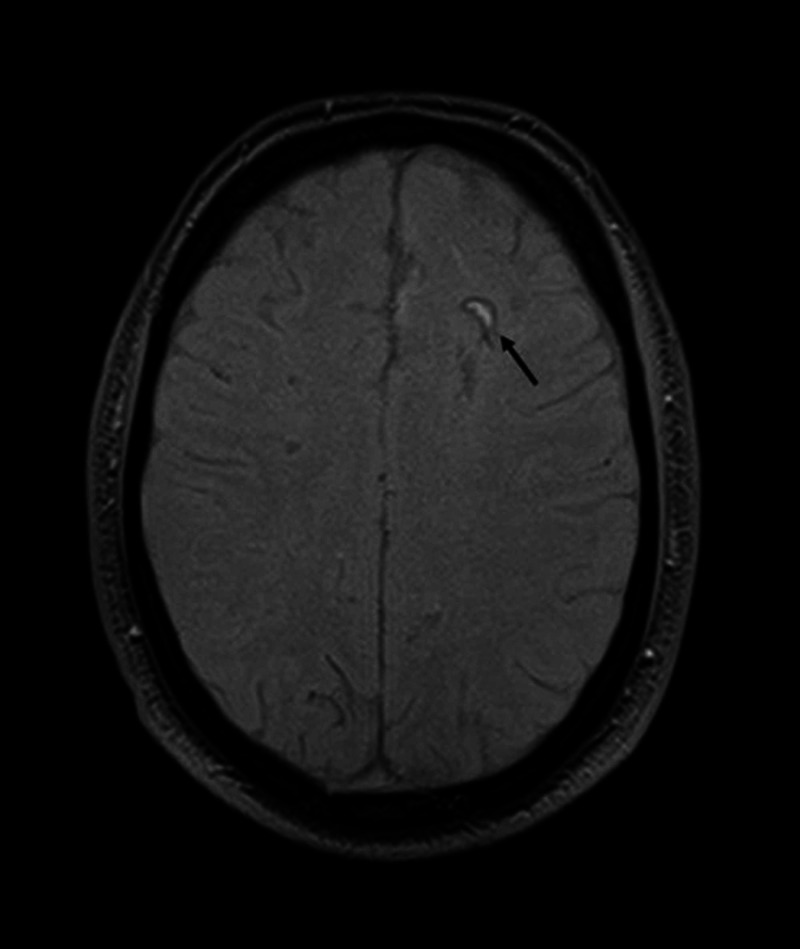
Hemorrhagic transformation of acute infarct on initial brain MRI The black arrow demonstrates a crescent-shaped hyper-intense area with peripheral susceptibility artifact in the left frontal lobe on the axial GRE sequence. Findings are considered consistent with the hemorrhagic transformation of the acute ischemic infarct. GRE - gradient echo

Computerized tomography (CT) brain showed small hypodense lesions in bilateral frontal regions without any mass effect and perilesional edema. Immediate magnetic resonance imaging (MRI) brain showed numerous multifocal extensive acute infarcts in bilateral frontal, and parietal lobes on diffusion-weighted (DW) and corresponding apparent-diffusion-coefficient (ADC) sequences demonstrating acute restricted diffusion (Figures 1-4). Few chronic infarcts with marginal gliosis were present in bilateral centrum semi-ovale (Figures 3-4). Acute infarcts in the left high frontal lobe demonstrated marginal susceptibility on the gradient-echo recovery (GRE) sequence, which was suggestive of the hemorrhagic component in infarct (Figure 5). Considering the initial possible diagnosis of multiple ischemic infarcts, the patient was started on standard antiplatelet therapy and lipid-lowering drugs, as the patient was not a candidate for thrombolytic therapy with tissue plasminogen activator (tPA): firstly, being out of the standard window period of 4.5 hours and secondly, the patient had a mild bleed which is a pure contra-indication for tPA.

**Figure 6:**
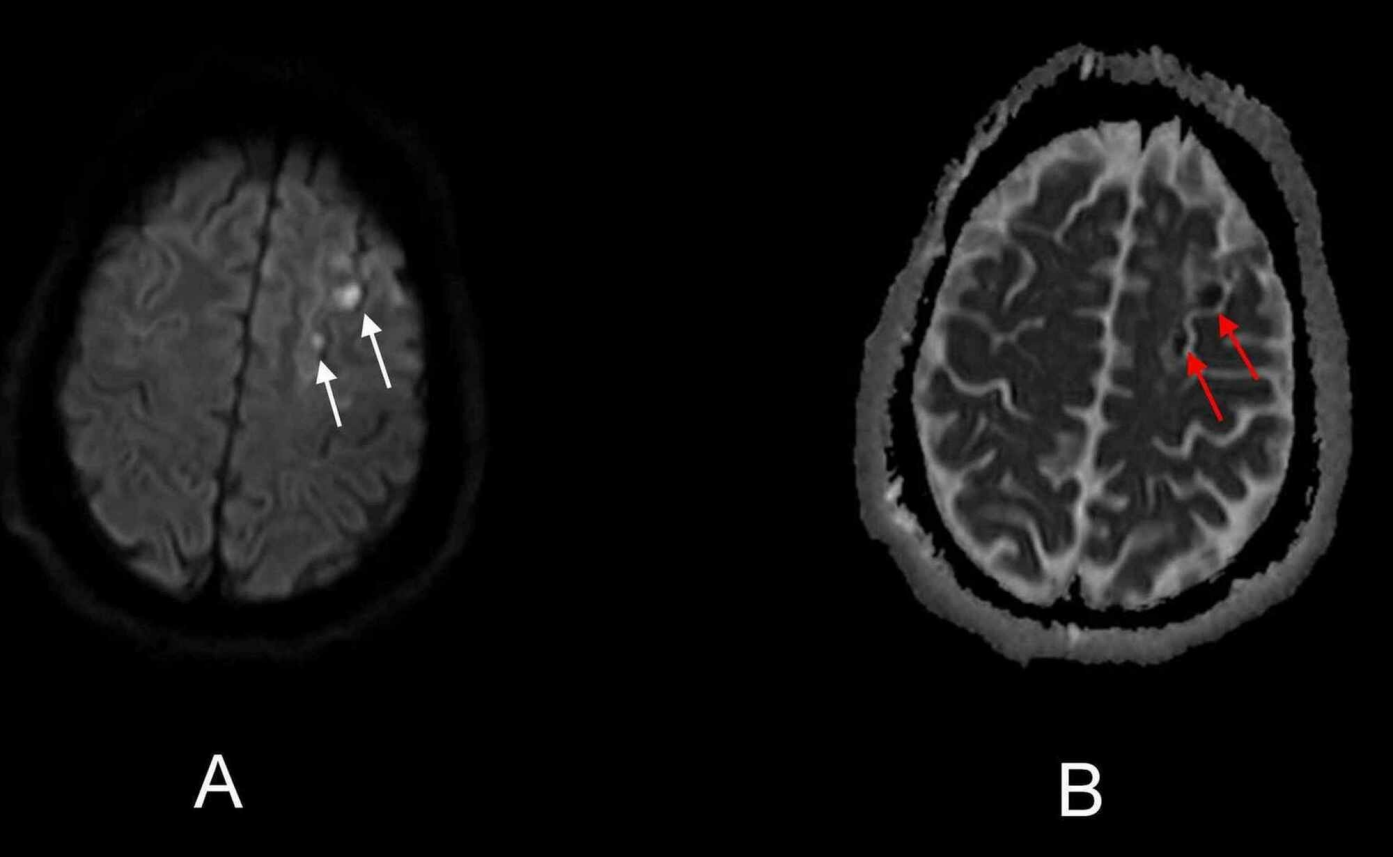
Axial DW and ADC sequence of the second brain MRI demonstrating new acute ischemic infarcts White arrows on axial DW sequence (A) and red arrows on axial ADC sequence (B) demonstrate two new acute ischemic infarcts in the left cerebral hemisphere manifested clinically as acute onset motor aphasia. DW - diffusion-weighted; ADC - apparent-diffusion-coefficient

**Figure 7:**
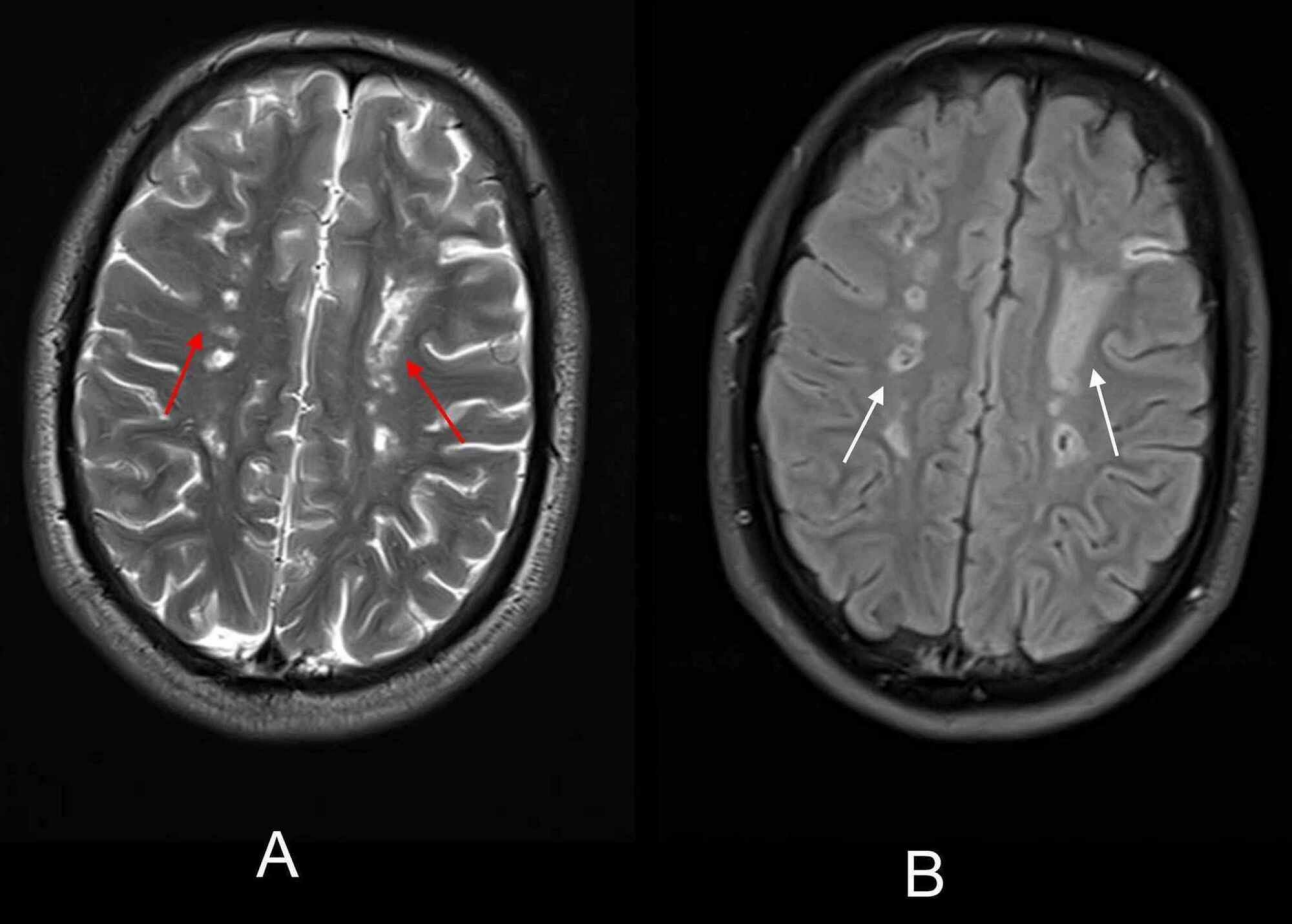
Axial T2-weighted and FLAIR sequence of the second brain MRI showing extensive chronic ischemic infarcts Red arrows on the axial T2-weighted image (A) and white arrows on FLAIR image (B) show multiple chronic ischemic infarcts in bilateral centrum semi-ovale and frontoparietal lobes. FLAIR - fluid-attenuated-inversion recovery

**Figure 8:**
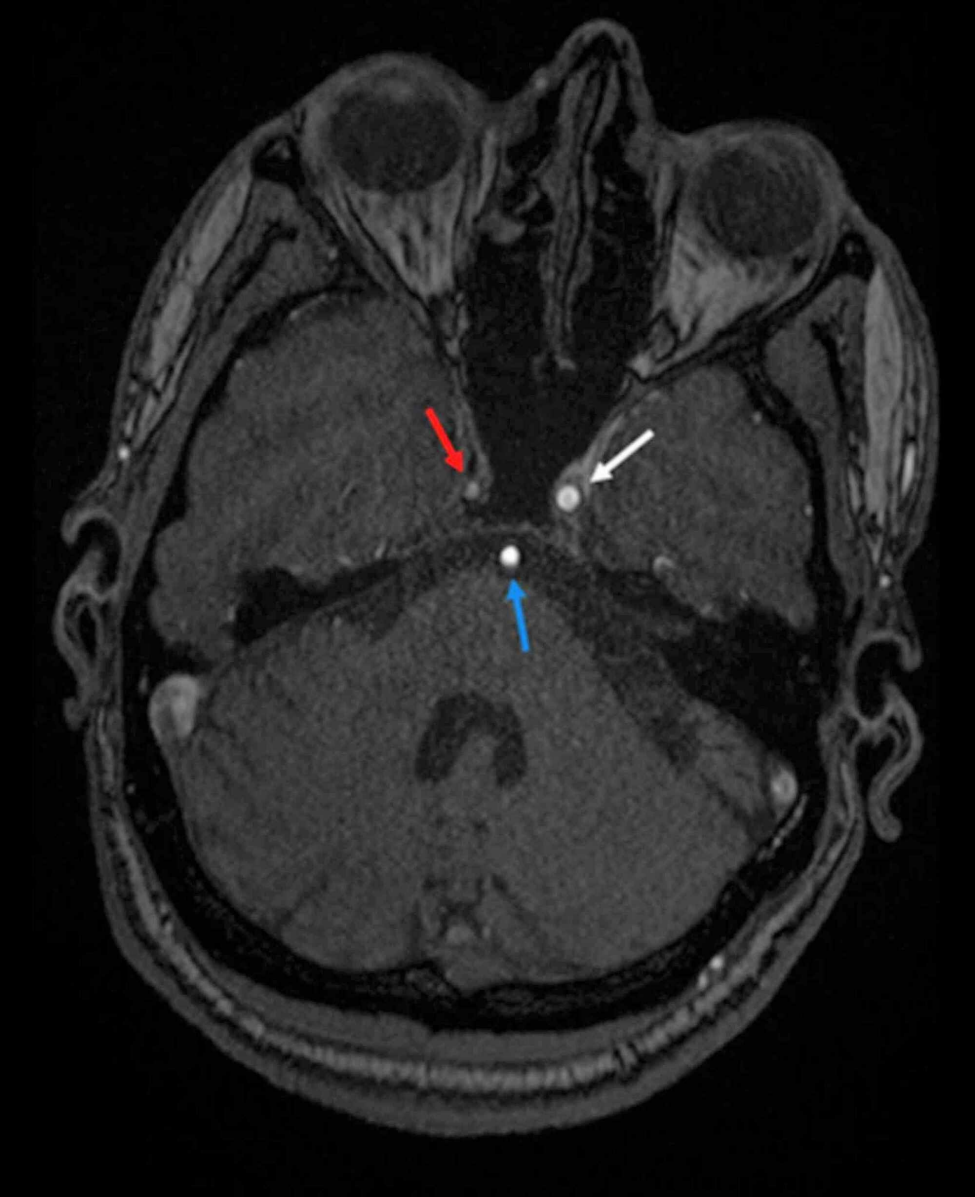
Axial MRA image of the brain The axial MRA image demonstrates the markedly attenuated caliber of the right ICA with faint opacification (red arrow). Left ICA (white arrow) and basilar artery (blue arrow) appear normal in caliber with normal contrast opacification. ICA - internal carotid artery

**Figure 9:**
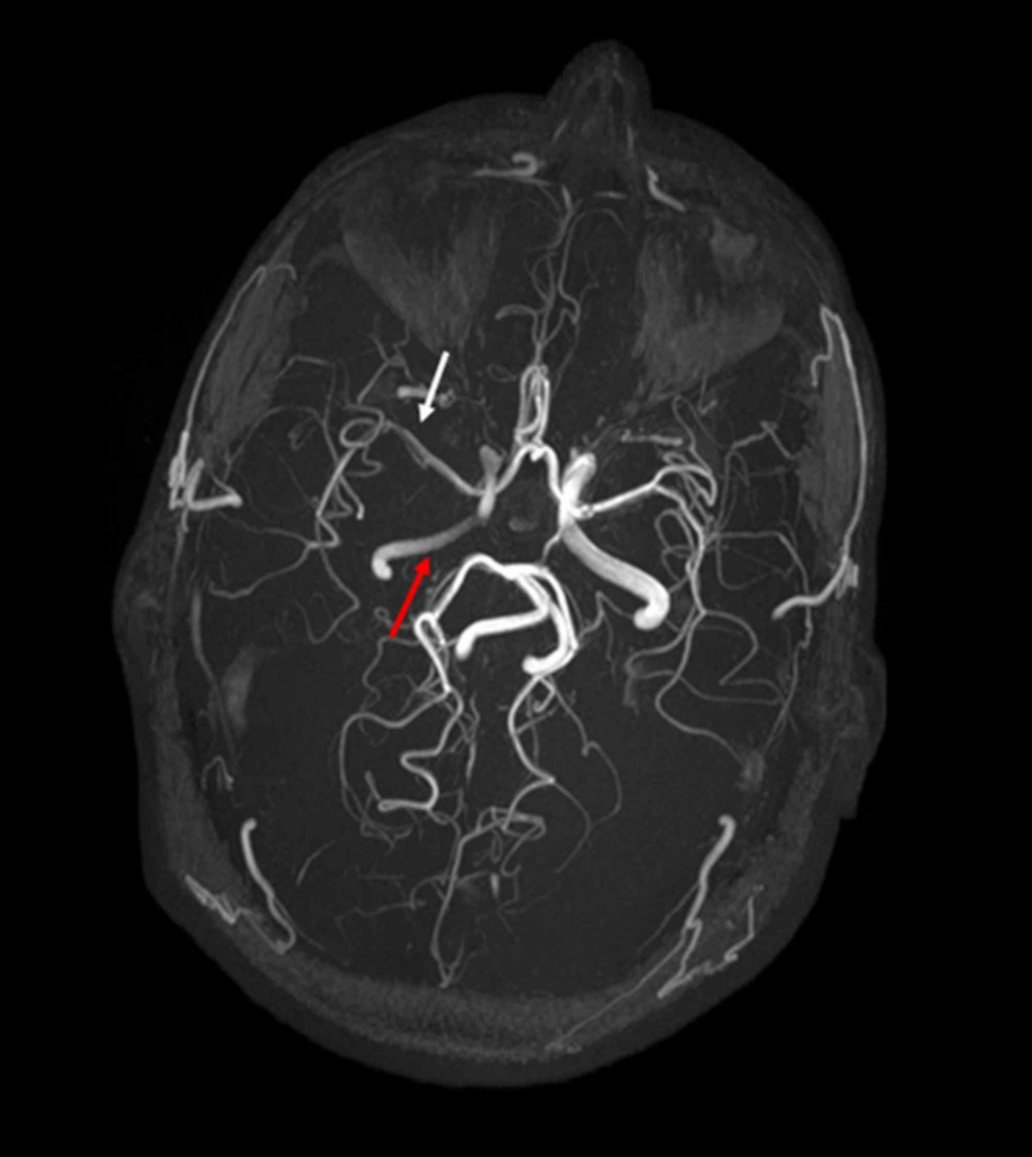
Axial MRA 3-D reformatted image of the brain Axial MRA 3-D reformatted image visualizes the intracranial arterial system. Right ICA appears attenuated in caliber (red arrows) involving its cavernous, clinoid, and supraclinoid segments. Subtle marginal irregularities (white arrow) are also noted in bilateral MCA and ACA. The right posterior communicating artery appears hypo-plastic (normal anatomical variant). ICA - internal carotid artery; MCA - middle cerebral artery; ACA - anterior cerebral artery

A month later, while the patient was still admitted and undergoing extensive investigations to find out any possible cause for multifocal ischemic infarcts, he developed complete motor aphasia during sleep. Again, an urgent MRI brain was carried out, which demonstrated two new small foci of acute ischemic infarcts in the left high frontal lobe evident by the restricted diffusion on DW and ADC sequences (Figure 6). Besides these acute infarcts, MRI also demonstrated previously documented, multifocal chronic infarcts with marginal gliosis in the bilateral centrum semi-ovale and bilateral frontoparietal lobes (Figure 7). Magnetic resonance arteriography (MRA) of the brain showed the attenuated caliber of the right internal carotid artery (Figures 8-9) with subtle marginal irregularities of bilateral anterior and middle cerebral arteries (Figure 9), possibly representing vasculitis.

Before developing motor aphasia, the patient had undergone many investigations, including cardiac Holter monitoring for a week, which did not reveal any electrical cardiac abnormality and echocardiography, which showed normal ejection fraction without any structural cardiac disease. Cerebrospinal fluid (CSF) had normal opening pressure, with raised proteins 55mg/dl, and no pleocytosis. CSF based polymerase chain reaction (PCR) testing for human-herpes virus I and II, cytomegalovirus, John Cunningham (JC) virus, Ebstein-Bar virus, and mycobacterium tuberculous bacillus was negative. Moreover, the cryptococcal antigen test was negative in CSF. Flow cytometry and immunocytochemistry did not reveal any abnormal cells in the CSF. Serological markers for hepatitis B virus, hepatitis C virus, human immunodeficiency virus, and treponema pallidum were non-revealing.

Complete thrombophilia screen was normal, including factor V Leiden variant, anti-thrombin III levels, protein C and S levels, and prothrombin gene mutation. C-reactive-protein (CRP) levels, erythrocyte sedimentation rate (ESR) were normal, and levels of angiotensin-converting enzyme (ACE) were within the normal range. The complete autoimmune screen was normal and included Anti-nuclear antibodies, ds-DNA antibodies, antibodies against an extractable nuclear antigen, anti-thyroid antibodies, anti-mitochondrial antibodies, anti-tissue trans-glutaminase antibodies, anti-endomysial antibodies, anti-smooth muscle kinase antibodies, and anti-phospholipid antibodies. The cluster of differentiation (CD) 55 and CD 59 deficiency was not present. Hemoglobin and serum protein electrophoresis did not show any hemoglobinopathy and immunoglobinopathy. Chest, abdomen and pelvis CT scan were also reported normal.

With this background, after second attack with motor aphasia patient underwent plasmapheresis sessions and was infused 1 g methylprednisolone intravenously daily for five days with possible suspicion of central nervous system (CNS) vasculitis; however, to confirm diagnosis further investigations like digital subtraction angiography (DSA) brain was performed. 

**Figure 10:**
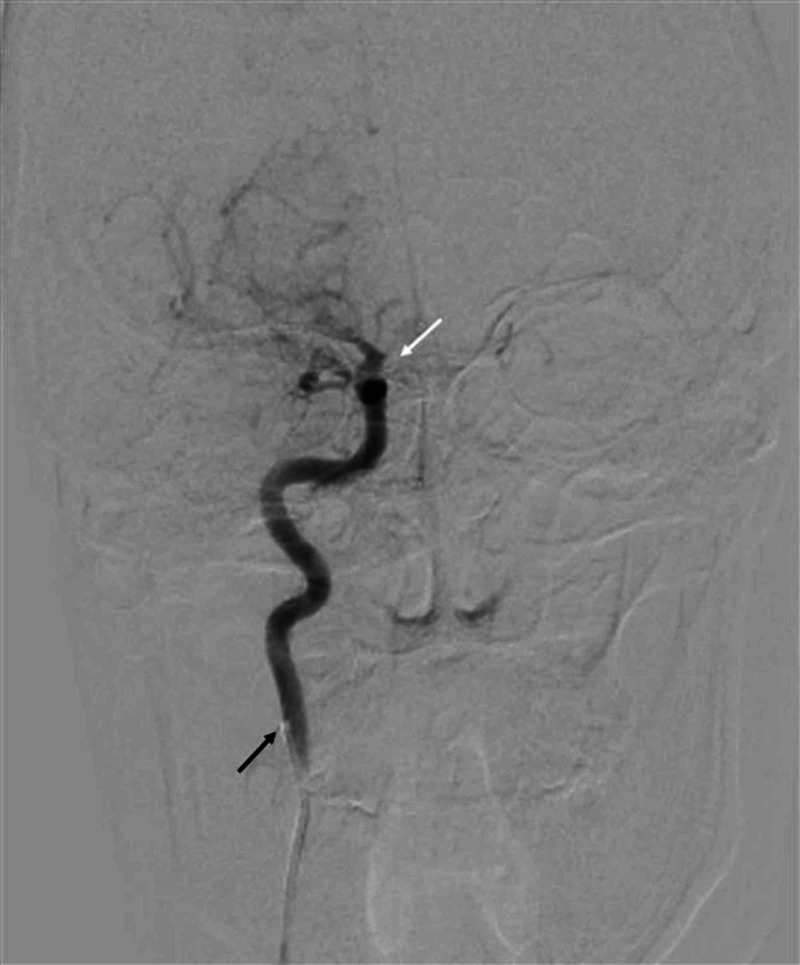
Right carotid artery angiogram, AP view on DSA brain showing complete occlusion of right ICA The catheter tip is visible in proximal right ICA (black arrow) with normal contrast flow in the proximal part; however, after giving an ophthalmic branch, the complete cut-off of contrast flow is evident in the right ICA (white arrow). AP - anteroposterior; DSA - digital subtraction angiography; ICA - internal carotid artery

**Figure 11:**
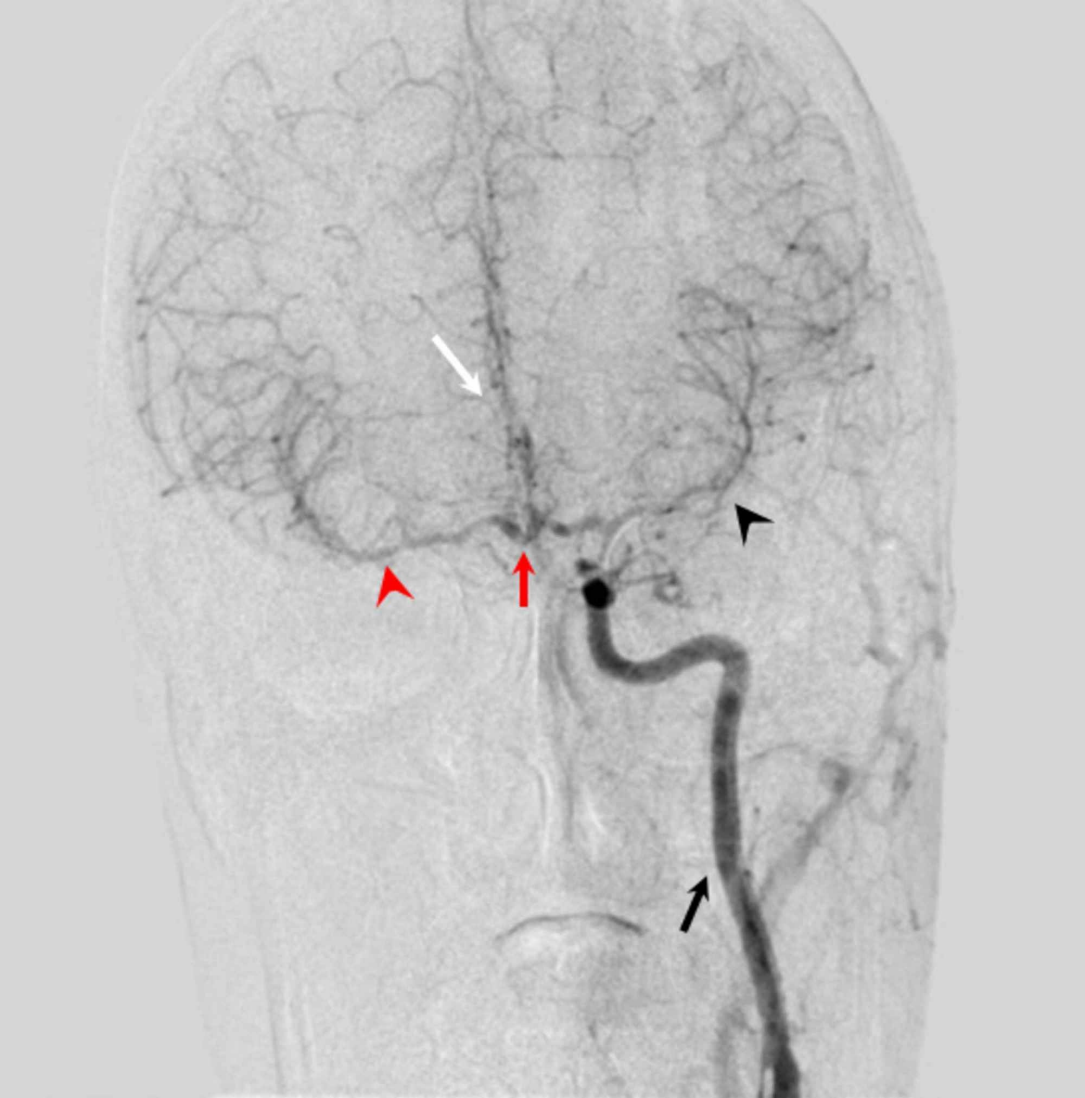
Left carotid artery angiogram, AP view on DSA brain showing anterior circulation of brain The catheter tip is visible in the left ICA with good contrast flow in the left ICA (black arrow). The right MCA (red arrowhead) and right ACA (white arrow) are being supplied from the anterior communicating artery (red arrow) indirectly from the left ICA. Left MCA (black arrowhead) and left ACA are directly supplied through left ICA. AP - anteroposterior; DSA - digital subtraction angiography; ICA - internal carotid artery; MCA - middle cerebral artery; ACA - anterior cerebral artery

**Figure 12:**
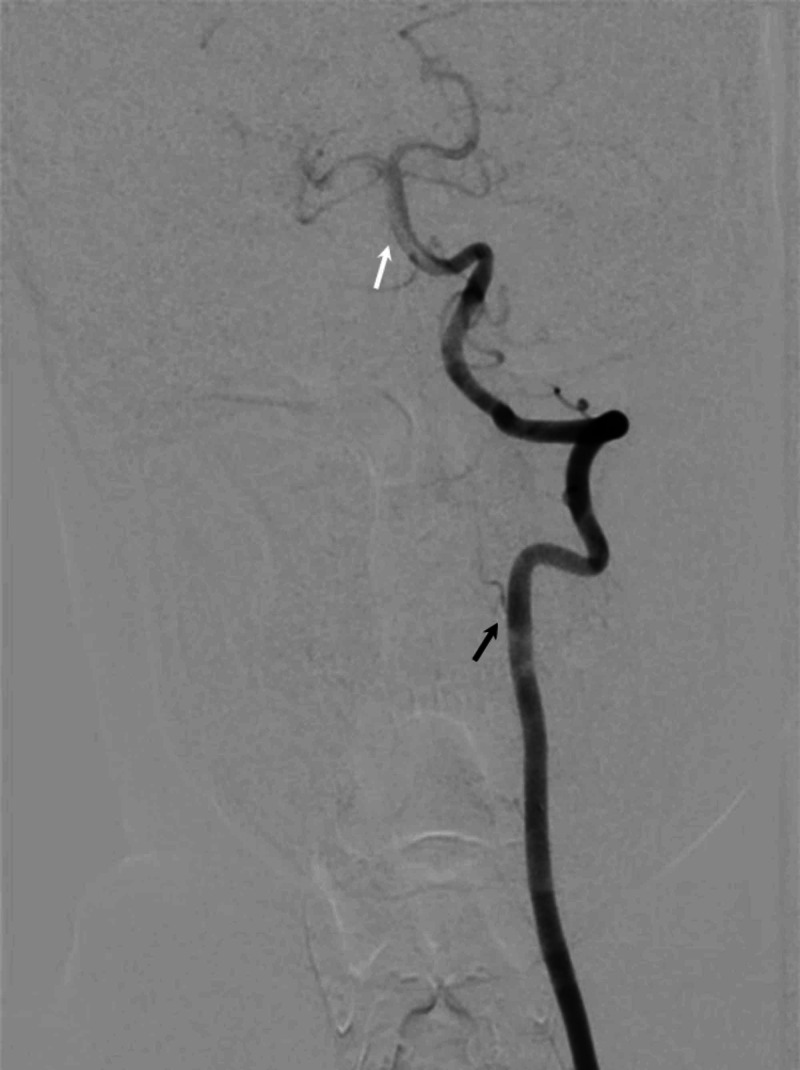
Left vertebral artery angiogram showing AP view on DSA brain depicting posterior circulation of the brain Normal contrast flow is visible in the left vertebral artery (black arrow) and basilar artery (white arrow). AP - anteroposterior; DSA - digital subtraction angiography

**Figure 13:**
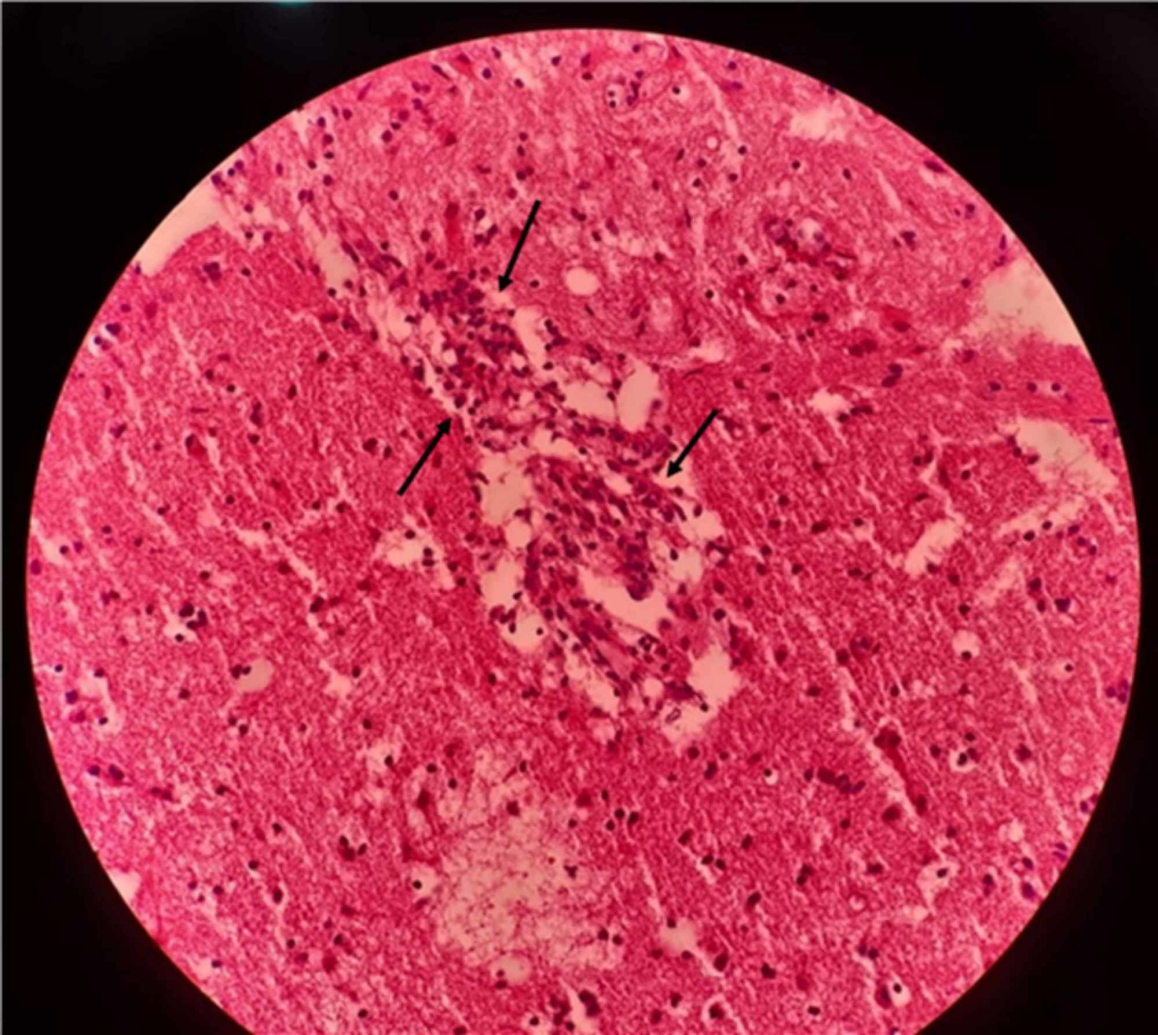
Biopsy specimen from left frontal lobe showing vasculitis The hematoxylin and eosin-stained section of the left frontal lobe lesion shows an area of infarction with a collection of gitter cells. Small blood vessels show endothelial swelling, intramural infiltration with small lymphocytes and perivascular lymphocyte cuffing (black arrows).

DSA brain showed a complete cut-off of contrast flow in the right internal carotid artery just after the origin of the right ophthalmic artery (Figure 10). The right middle cerebral artery (MCA) and anterior cerebral artery (ACA) were supplied by the left internal carotid artery through the anterior communicating artery (Figure 11). Bilateral vertebral and basilar artery were normal in caliber without any irregularity (Figure 12); however, right-sided MCA showed irregularities to be consistent with vasculitis. A multi-disciplinary meeting was held, and it was decided to perform a brain biopsy. Histopathological specimens taken from the left frontal lesion showed chronic inflammation with mild perivascular inflammation (Figure 13). Moreover, the Congo-red stain did not reveal amyloid deposits. Immunohistochemistry showed the positivity of CD68 in gitter cells and CD3 in most lymphocytes, CD20 on B cell lymphocytes, while CD34 was positive in blood vessels.

These findings were considered due to vasculitis but were mild, probably due to the partial treatment with intravenous steroids three weeks prior to the brain biopsy. The final diagnosis was primary CNS vasculitis, and the patient was started on rituximab 1 g two doses two weeks apart every six months. In one year, the patient was able to walk independently without any support. He was undergoing regular sessions with speech therapists for aphasia, which had improved only minimally. 

## Discussion

PACNS has a usual clinical presentation ranging from insidious symptoms with a subacute or chronic course of the disease to patients presenting acutely with only a few days since symptom onset [5, 6]. Our patient had an accelerated progression of the disease with the development of a second stroke one month into the admission that resulted in complete motor aphasia. In about 60% of patients diagnosed with PACNS, headache is evident at presentation, which gives a hint to physicians of the likely diagnosis [1, 2]. Absence of headache may lead to PACNS not getting high order of priority in the list of differential diagnoses in a patient presenting with the acquired neurological deficit as in our patient. 

PACNS differs from secondary CNS vasculitis in that it usually doesn't have constitutional symptoms such as fever, weight loss, night sweats. ESR and CRP are usually within the normal range [7]. There are no serological markers that indicate the presence of PACNS; those done are used to rule out other etiologies.The presence of cerebral venous sinus thrombosis indicates a systemic cause of vasculitis, such as Behcet's syndrome rather than PACNS [8].

The diagnosis of PACNS is made primarily by the presence of acquired neurological deficit; exclusion of infectious, neoplastic, systemic causes; and documentation of either classic angiographic and/or histopathological findings of angiitis of CNS [9]. Ruling out other etiologies, obtaining brain biopsy, and performing angiography may delay diagnosis, which due to the disease variable rate of progression, can result in poor patient outcomes. A new screening tool has been proposed by Sarti et al. for highlighting those patients who might benefit from early diagnostic tests [2]. Applying this retrospectively to our case at the time of initial presentation to the hospital, our patient fulfilled the criteria of the screening tool; this may have changed the clinical course of the patient had DSA and brain biopsy been performed early with the prompt institution of treatment.

Our case highlights that prophylactic therapy may also be started, especially in young patients with other etiologies such as atherosclerosis are less likely after ruling out an infection. This would prevent further ischemic insults from happening as investigations are being carried out in a patient. Treatment of PACNS is based primarily on glucocorticoids and cyclophosphamide. Rituximab has also been used to treat PACNS [3, 10].

## Conclusions

PACNS is an elusive disease presenting a challenge to clinicians in diagnosing and treating the condition promptly. The course of the disease can be varied significantly with the prompt institution of therapy. The predicament lies in determining whom to suspect and carry out diagnostic investigations thus improving patient outcomes in this rare disease. The development and validation of new screening tools are essential to picking up suspected cases of PACNS early as the progression of the disease can be quickly leading to disability which might be prevented with swift diagnosis and treatment.
